# Optical Oxygen Sensors for Applications in Microfluidic Cell Culture

**DOI:** 10.3390/s101009286

**Published:** 2010-10-15

**Authors:** Samantha M. Grist, Lukas Chrostowski, Karen C. Cheung

**Affiliations:** Department of Electrical & Computer Engineering, University of British Columbia/2332 Main Mall, Vancouver, BC V6T 1Z4, Canada; E-Mails: lukasc@ece.ubc.ca (L.C.); kcheung@ece.ubc.ca (K.C.C.)

**Keywords:** optical oxygen sensors, luminescence, microfluidics, cell culture, lab-on-a-chip

## Abstract

The presence and concentration of oxygen in biological systems has a large impact on the behavior and viability of many types of cells, including the differentiation of stem cells or the growth of tumor cells. As a result, the integration of oxygen sensors within cell culture environments presents a powerful tool for quantifying the effects of oxygen concentrations on cell behavior, cell viability, and drug effectiveness. Because microfluidic cell culture environments are a promising alternative to traditional cell culture platforms, there is recent interest in integrating oxygen-sensing mechanisms with microfluidics for cell culture applications. Optical, luminescence-based oxygen sensors, in particular, show great promise in their ability to be integrated with microfluidics and cell culture systems. These sensors can be highly sensitive and do not consume oxygen or generate toxic byproducts in their sensing process. This paper presents a review of previously proposed optical oxygen sensor types, materials and formats most applicable to microfluidic cell culture, and analyzes their suitability for this and other *in vitro* applications.

## 1. Introduction

### 1.1. Oxygen and Cells

Oxygen is an immensely important species in biological systems. Molecular oxygen plays a crucial role in the behavior and viability of many types of cells as well as the properties of human tissues [1]. Although the atmospheric oxygen level in air is 21%, the normal level in the human alveoli is 14%. This level decreases away from the blood vessels and forms an oxygen gradient in many tissues, with normal levels varying from organ to organ [2]. Hypoxia, or inadequate oxygen levels, has a large effect on cells and tissues, including inducing vasodilation [3] and changing metabolic processes to reduce oxygen consumption [4]. Tissue hypoxia in cancerous tumors has been linked with resistance to radiation therapy and many anticancer drugs [5], as well as increased likelihood of metastasis and decreased likelihood of patient survival and treatability [6,7]. Oxygen levels in tumors are often significantly lower than those in normal tissues [5,6], leading to the development of hypoxia-activated anticancer drugs designed to specifically target the hypoxic tumor tissues [6].

Oxygen level has also been identified as an important parameter in stem cell cultivation and differentiation. Stem cell proliferation can be enhanced and apoptosis reduced in cultivation conditions with oxygen levels lower than the standard 20% [8]. Changes in stem cell cultivation environment oxygen concentration can also be used to simulate *in vitro* the effects of disease [8]. Stem cell differentiation patterns are also highly dependent on oxygen levels [8,9]. Embryonic development often occurs in low-oxygen environments, and oxygen has been found to be an important signal molecule to regulate stem cell differentiation. As such, carefully controlling the oxygen concentrations in stem cell populations *in vitro* is essential for controlling the cells’ differentiation and maintaining undifferentiated populations [9]. In regenerative medicine, the transplantation of new stem cells may be used to replace cells which have been lost through disease or injury. Understanding the dynamic oxygen conditions during normal tissue development will be necessary to control differentiation or apoptosis of stem cells. Oligodendrocyte progenitor cells, which may be used for the treatment of demyelinating diseases, should be initially cultured in 5% O_2_ and then differentiated in 20% O_2_ for increased cell production [10]. These conditions should be reproduced in the production of cells for replacement therapies.

Because of the profound effect oxygen has on biological systems, controlling and monitoring oxygen concentrations is useful in many cell culture applications. Consequently, there has been much interest in the development of inexpensive oxygen sensors and control mechanisms that can be easily integrated with cell culture environments. In addition to the simple oxygen-sensing application, oxygen sensors can also be adapted for the measurement of glucose concentrations through the addition of glucose oxidase, which allows glucose levels to be determined from oxygen levels because an amount of oxygen dependent on the glucose concentration is consumed in the oxidation of glucose by glucose oxidase [11–14]; this further increases the applicability of oxygen sensors.

### 1.2. Microfluidics for Cell Culture and Cell-Based Studies

Microfluidics involves sub-millimeter-scale fluidic channels and their application to a wide variety of problems in biology, chemistry, and other areas. The small size-scale of microfluidic channels yields a number of advantages over the traditional methods used in these areas. The small fluidic volumes lead to lower reagent costs [15]. Furthermore, the microfluidic chips themselves are often fabricated from inexpensive polymers [16,17] and can also be mass-produced. The small fluidic volumes also reduce the time it takes for reactions to be carried out and afford reduced heat transfer times [18].

The application of microfluidics to cell-based research appears to be particularly promising. Microtechnology has been used to fabricate structures for almost every step in the cell research process: cell acquisition; cell culture, trapping, and sorting; cell treatment; and finally analysis [19,20]. Microfabrication and microfluidics are ideal for working with cells as the structures present within them are on the same size scale as the cells themselves [19]. This size compatibility facilitates greater control over the cells’ position and the cell culture environment. In addition, microstructures present in microfluidic devices can provide a 3-D cell culture environment which more closely emulates the natural cell growth conditions than traditional 2-D cell culture environments [21]. Moreover, microfluidics can be used to create biomolecular gradients, which are important for guiding cell growth, migration, and differentiation within tissues. Microflow control permits precise routing of fluids in order to create predictable and reproducible gradients at the microscale, allowing us to better study these biological phenomena. Microfluidic gradient generators have been used to create gradients in signaling proteins for the study of chemotaxis, immune response, cell differentiation, and cancer [22]. Finally, microscale devices are ideal for studies involving small cell populations, such as primary cancer cells obtained from needle biopsies, or stem cells.

A number of interesting reviews summarize the progress made in the application of microfluidics to biology [15] and more specifically, cell-based research and cell culture [19–21,23,24]. In many cases, the eventual goal for microfluidic systems is to create “lab-on-a-chip”-type microfluidic devices, which integrate all of the necessary steps for analysis onto a single chip [25,26]. Lab-on-a-chip systems also promise to have a large impact in cell-based drug testing and drug discovery [18]. For high-throughput screening in cell-based assays, microflow control can give high precision in fluid handling, leading to high pipetting reliability and good cell seeding uniformity over large numbers of wells [27]. This is important for cell-based assays since the readout depends on the cellular response. The increased automation possible with microfluidic systems allows reagents and nutrients needed for cell growth to be supplied and the cells’ waste products to be removed in a more controlled and reproducible manner than that often found in traditional cell culture technologies [18,19,21]. One of the ways in which “lab-on-a-chip” devices aim to integrate a whole lab’s worth of functionality into a microfluidic device is by including sensing functionality in the chip itself.

Integrating sensors and detectors within microfluidic channels reduces the need for external infrastructure such as analyte vessels to take measurements from the device [25]. More importantly, incorporating sensors inside the microfluidic channel permits direct *in situ* measurements, as the data is recorded at the time of interest rather than after the fluid has exited the channel. As it is often desirable to accurately monitor various parameters in the cell culture environment, there has been an effort to integrate many types of sensors into microfluidic channels for cell culture, including dissolved oxygen and carbon dioxide [28], pH [29,30], and temperature [31,32]. Dissolved oxygen sensing in particular has generated much interest, and as such will be the focus of this review.

### 1.3. Oxygen Sensors

Much of the early work on oxygen sensors focused on Clark-type electrode sensors [33], which detect a current flow caused by reduction of oxygen. Such sensors have been miniaturized and integrated with microfluidic devices to monitor the oxygen consumption of bacteria [34]. The miniaturization of such devices requires microscale electrodes, and this type of sensor consumes oxygen (and thus requires sample stirring for accurate measurements), is easily contaminated by sample contents, and requires electrical connection between the sensor electrodes and the measurement infrastructure [35]. These factors present several significant disadvantages for microfluidic cell culture systems.

Consequently, there has been much interest in the integration of optical oxygen sensors with microfluidic systems. These optical sensors present the advantages that they are easily miniaturized, are not easily contaminated, do not require physical contact between the sensor and optical detector, and do not consume oxygen [12,35–39]. Most optical oxygen sensors operate on the principle of reversible luminescence quenching of the intensity or excited-state lifetime ([40], as cited in [41]) of a luminescent indicator dye or luminophore. This process occurs when the excited state energy of a fluorescent or phosphorescent indicator molecule is transferred to another molecule such as oxygen rather than being emitted in the form of a luminescence photon [42]. The quenching behavior can be modeled by the Stern-Volmer equation [43]:

(1) $$\frac{\tau_{0}}{\tau }=\frac{I_{0}}{I}=1+k_{Q}\tau_{0}pO_{2}$$

where *pO_2_* is the partial pressure of oxygen, *k_Q_* is the quenching rate constant, *τ*_0_ and *I*_0_ are the excited-state lifetime and luminescence intensity in the absence of oxygen, respectively, and *τ* and *I* are the excited state lifetime and luminescence intensity at the pressure of interest, respectively. The Stern-Volmer equation may also be written in terms of the dissolved oxygen concentration [*O_2_*] rather than *pO_2_*, requiring different units for *k_Q_*.

There are several excellent reviews of optical oxygen sensors [37,44], as well as more general optical sensors [14,45] and oxygen sensors [35]. This paper aims to both present relevant work on optical oxygen sensors and analyze the methods’ compatibility with microfluidic cell culture.

There are many ways in which to classify the previous work on optical oxygen sensors, and a great many sensor designs have been proposed. In Section 2 of this paper, the two main optical oxygen-sensing methods (based on the luminescence intensity and excited-state lifetime as in the Stern-Volmer equation) will be discussed. Section 3 will present some of the commonly used indicator molecules and summarize some of the work in which they have been used. In addition to these two factors, the sensing molecule is often encapsulated in an immobilization material to prevent its unwanted interaction with the sensing environment (for example, inducing toxicity or becoming less sensitive to oxygen as a result of interaction with the environment or biological materials). Section 4 will summarize some of these immobilization materials previously used for optical oxygen sensing, while Section 5 will discuss the different formats previously used for optical oxygen sensors. Section 6 will present the optical measurement systems used to supply the excitation light and detect the luminescence. In addition to presenting the previous work in each of these areas, each section will evaluate the different methods’ suitability for microfluidic systems. Finally, Section 7 will present some of the previous work integrating optical oxygen sensors with microfluidic cell culture and Section 8 will conclude the review with a summary and description of future outlook in this field.

## 2. Optical Oxygen Sensing Methods

Optical, luminescence-based oxygen sensing is based on the phenomenon of luminescence quenching by oxygen. As oxygen quenches both the luminescence intensity and excited-state lifetime, there are inherently two different methods of measuring oxygen concentrations or pressures with luminescent probes. This section will present some of the previous work performed using each method, outline the methods’ advantages and disadvantages, and evaluate their compatibility with microfluidic systems.

### 2.1. Detection of Luminescence Intensity

**Figure 1.**
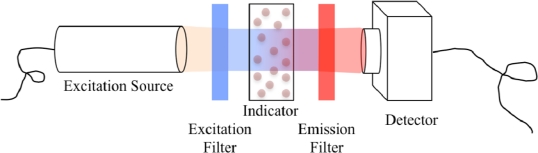
Simplified example setup for intensity-based optical oxygen sensing.

Intensity-based oxygen sensing involves only the detection of the luminescence intensity, and as a result is generally easier to implement than lifetime-based detection methods. An example setup for intensity-based detection is presented in Figure 1.

The luminophore is excited by light from an excitation source, which passes through an excitation filter to select the wavelengths best matched to the excitation spectrum of the luminophore. The emitted luminescence intensity is detected after passing through an emission filter to remove any extraneous light not part of the emission spectrum. A detector array such as a Charge-Coupled Device (CCD) can easily be used to detect the emitted luminescence, allowing 2-D oxygen concentration gradients to be determined. The simplified setup depicted in Figure 1 does not include any imaging optics, but lenses [46] and even complete fluorescence microscopy setups [39,47,48] can be easily integrated into the intensity imaging setup.

Intensity-based sensing suffers from several disadvantages, including susceptibility to photobleaching, leaching, and intensity variations caused by inhomogeneities in the detector pixels (if a 2-D detector is used); dependence on detection optics, sample absorption or scattering, excitation light, and dye layer concentration and film thickness [38]. Nevertheless, intensity-based imaging has been successfully used for *in vivo* sensing applications [47,49], gaseous oxygen sensing [50], inter- and intra-cellular measurements [51,52], and microfluidic oxygen sensing [48,53–55].

Intensity-based measurements are particularly attractive for microfluidic cell culture because of their inherent compatibility with standard fluorescence microscopy setups often already in place and because of the simplicity of the measurement method. Several methods have also been proposed to help overcome the disadvantages of intensity-based sensing. The best-investigated method has been ratiometric sensing [39,51,56,57], wherein the sensing layer contains both the oxygen-sensitive dye and an oxygen-insensitive dye, with the two dyes having different emission spectra. Both dyes are excited by the excitation source and the sum of the two emission spectra is detected by a detection spectrometer, but only the emission intensity of the oxygen-sensitive dye is quenched by the presence of oxygen. The oxygen levels are thus determined by measuring the ratio between the emission intensities of the two dyes. This method helps reduce the effect of excitation light, dye layer, detection optics, detector sensitivity, and sample inhomogeneities, as the emission intensity of the oxygen-insensitive dye is also affected by these factors. Other methods used to improve the accuracy of intensity-based sensing have included the formulation of complex calibration functions incorporating photobleaching and leaching effects and pixel-by-pixel calibration techniques [47] requiring no sample movement between calibration and sensor use.

Despite these efforts to improve intensity-based luminescent oxygen sensing methods, several groups have concluded that lifetime-based optical oxygen measurements (discussed below) are superior to and more robust than intensity-based measurements [38,58–61] using the same probe molecules. Detection methods based on phosphorescence lifetime also yield improved contrast and suppression of background signal [46]. As such, much of the recent work on luminescent oxygen sensors has focused on lifetime-based sensing methods.

### 2.2. Detection of Luminescence Lifetime

**Figure 2.**
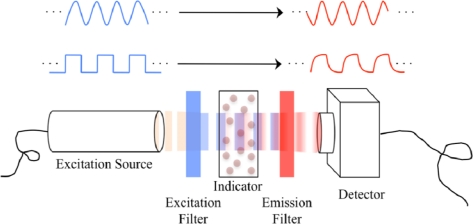
Simplified example setup for lifetime-based optical oxygen sensing. Example excitation modulation and emission waveforms are also shown.

Lifetime-based sensing mechanisms involve the detection of the luminescence lifetime in either the time domain or the frequency domain. Time domain detection generally involves the direct detection of the lifetime itself, while frequency domain detection generally involves determining the luminescence lifetime via a lifetime-dependent phase lag between the excitation and emission light intensity waveforms. For both lifetime-based sensing mechanisms the excitation illumination must be modulated. A simplified example setup for lifetime-based oxygen sensing is shown in Figure 2. Also included in Figure 2 are example excitation light modulation and their corresponding emission waveforms. The sinusoidal excitation modulation waveform likely corresponds to a phase-based detection method, wherein the fluorescence lifetime affects the phase shift between excitation and emission sinusoids. Conversely, the square-wave excitation modulation waveform corresponds to a time-domain detection mechanism.

**Figure 3.**
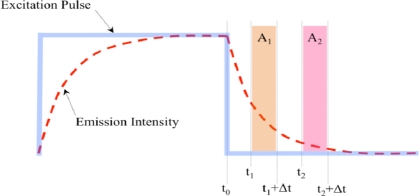
Illustration of “pulse-and-gate” time-domain luminescence lifetime detection. The transparent colored boxes indicate the windows of data acquisition; the decay constant and luminescence lifetime can be determined from the data acquired in these windows (figure adapted from [28]).

The most common time-domain lifetime detection scheme is the “pulse-and-gate” method [28,62–68], as illustrated in Figure 3. In this method, the excitation light is modulated (generally by a square-wave pulse indicated by the thick blue line) and the detector is gated such that it acquires windows of emission intensity data (indicated by the colored regions), generally during the luminescence decay period. The dashed red line represents the intensity of the emitted light.

Two acquisition windows are sufficient to characterize a monoexponential decay and are commonly used [28,62,63], although three-window and even five-window methods have been used for improved accuracy [46,66]. The ratio of the integrated data collected during the two windows can be used to determine the decay constant of the signal and thus the luminescence lifetime of the indicator, via Equation 2 [65]:

(2) $$\tau =\frac{t_{2}-t_{1}}{\text{ln}\frac{A_{1}}{A_{2}}}$$

With the “pulse-and-gate” method, it is possible to remove the effects of short-lived background luminescence and any residual, decaying source light after the nominal shutoff time. This is usually accomplished by adding a short delay (∼100–500 ns) between the end of the excitation pulse and the beginning of the first gated window [28,63]. It is much more difficult to separate background luminescence with long lifetimes or similar lifetimes to that of the indicator of interest [46]. The “pulse-and-gate” method of lifetime detection has been successfully used with oxygen-sensitive indicators and gated detectors to obtain two-dimensional oxygen distribution images in micro-titer plates [28], engineered tissue, living cells, and *in vivo* samples [62,66,69,70], coral sediment, lichen, and foraminifer samples [63], microfluidic bioreactors [70,71], and biofilm growth flow chambers [72]. As long as the detector only detects the luminescence signal while the excitation lamp is not emitting and the effects of ambient light are insignificant, the emission filter shown in Figure 2 is not necessary for time-domain lifetime detection. For other methods such as phase-based lifetime detection, however, it is necessary to include the filter.

Another time-domain method of measuring luminescence lifetime involves taking the ratio of gated detection windows different from those illustrated in Figure 3 [38]. This method utilizes one window during the excitation pulse and another after the pulse, and the ratio of these windows (after subtracting any effects of dark current) may be used to determine the luminescence lifetime. This detection scheme has been compared to the “pulse-and-gate” method, and found to have a higher signal-to-noise ratio and faster calculation time [46] due to the longer windows and increased optical power during each window. Its disadvantages include its inability to separate out background luminescence and the need for an emission filter.

The frequency-domain method of determining luminescence lifetime (phase fluorometry or luminometry) measures the phase shift between the excitation light intensity and emitted light intensity waveforms. If the luminescence decay is modeled as single-exponential, the luminescence lifetime *τ* may be obtained from the phase shift *ϕ* using Equation 3 [73]:

(3) tan(φ)=ωτ

where *ω* is the angular frequency of modulation. The optimal modulation frequency for frequency-domain lifetime measurements may be found from τ_1_ and τ_2_, the lifetimes (*i.e*., quenched and un-quenched) of interest, using Equation 4 [59]:

(4) $$\omega_{\mathrm{opt}}=\frac{1}{\sqrt{\tau_{1}\tau_{2}}}$$

Frequency-domain methods of lifetime detection require detection mechanisms capable of detecting phase differences, but separation of luminophores with close lifetimes is easier than with time-domain methods [46]. Phase fluorometry or luminometry was first used with simple point detectors such as photodiodes and photomultiplier tubes (PMTs) [74–76] but has also been expanded to use two-dimensional detectors to obtain two-dimensional images of oxygen distributions [77]. Phase-based optical oxygen sensing with photodiode detectors has also been successfully integrated into microfluidic channels and bioreactors [78–80] and even multi-chamber microfluidic cell culture analog systems [81].

## 3. Oxygen-Sensitive Luminescent Materials

The sensitivity and other properties of optical oxygen sensors are dependent on a number of factors, most importantly the luminophore, or luminescent indicator. There are several properties to be considered when choosing the optimal indicator for a certain application. One of the most important properties of a luminescent indicator is how readily its emission is quenched by oxygen. This factor is dependent on the efficiency of the quenching process itself as well as the excited-state lifetime of the indicator, as the probability of the indicator interacting with oxygen increases when electrons are in the excited state for a longer time period [37]. For the sensor to be usable over long time periods and even be reusable, the indicator should be stable against photobleaching and leaching into the tested sample. The absorption and emission spectra of the dye are also often considered in the selection of luminescent indicators. It is often desirable for these spectra to be compatible with inexpensive and readily available excitation sources, detectors, and filters. Additionally, some materials (such as human plasma [56] and mammalian cells [82]) autofluoresce and this confounding signal can be removed either by the use of an emission filter or a lifetime detection method with good lifetime selectivity (such as frequency domain lifetime detection) after selection of materials/indicators with a sufficiently different emission spectrum or luminescence lifetime. Alternatively, materials with different excitation spectra from those of the autofluorescent materials may be selected to overcome this problem.

Various oxygen-sensitive indicators have been identified and used for various applications. Many of these indicator compounds fall into two main groups: ruthenium-based molecules or metallo- porphyrin-type molecules. Other, less commonly used, oxygen-sensitive compounds include fluoresce in compounds [83], polycyclic aromatic hydrocarbons [42], and other organic compounds [44].

The following sections will introduce some of the most commonly-used oxygen-sensing compounds and discuss their applicability to microfluidic cell culture. More general reviews of oxygen-sensing compounds may be found in [44], and a review of various phosphorescent metallo-porphyrin complexes and their applications (not limited to oxygen sensing) is presented in [84].

### 3.1. Ruthenium-based

Several fluorescent, ruthenium-based compounds have been applied to optical oxygen sensing. Compounds of ruthenium-tris-4,7-diphenyl-l,l0-phenanthroline ([Ru(dpp)_3_]^2+^) [13,36,41,46,59,60,63,77,81,85–90] and ruthenium(II)-tris(l,l0-phenanthroline) ([Ru(phen_3_]^2+^) [28,38,47,91] are commonly-used examples, and they have been modified to be soluble in silicone films for oxygen sensing [92]. Other ruthenium compounds used in optical oxygen sensors include dichlorotris (1,10-phenanthroline) ruthenium (II) hydrate [93] and ruthenium tris (2,2′-dipyridyldichloride)hexahydrate [50,64,66,71,94,95].

Oxygen-sensitive, fluorescent ruthenium compounds have been used extensively in optical oxygen sensing and have even been previously integrated with microfluidic bioreactors and other devices [71,81,94]. While the ruthenium complexes have a high luminescence quantum yield and are very photostable, their short excited-state lifetimes (on the order of 100 ns–1 μs [96]) lead to lower sensitivity to oxygen than is necessary in certain applications. These applications are in low-oxygen environments (e.g., modified-atmosphere food packaging with oxygen partial pressures of 0–2 kPa [90], and culture of anaerobic bacteria with dissolved oxygen levels less than 12 ppm [55]), which necessitate highly sensitive oxygen sensors, and alternative oxygen-sensitive compounds such as some of the metalloporphyrin-type indicators fill this requirement. Most metalloporphyrin-type indicators phosphoresce rather than fluoresce, which leads to a lower luminescence quantum yield but a longer excited-state lifetime (on the order of 10 μs–1 ms [37]) and thus a higher sensitivity to oxygen.

### 3.2. Metalloporphyrin-based

Platinum(II)– and palladium(II)– complexes of octaethyl–porphyrin (Pt- and Pd- OEP) [97,98] have been used successfully in optical oxygen sensors for *in vivo* applications [91], engineered tissues [62], aquatic sediments [46,59,60,87], microtiter plates [28], intracellular applications [51], and other biological applications [52]. They demonstrate a long luminescence lifetime and high quantum yield but relatively poor photostability, inhibiting their use in many applications.

Platinum(II)- and palladium(II)- complexes of octaethyl–porphyrin ketone (Pt- and Pd- OEPK) [61] were introduced as another set of potential phosphorescent sensing dyes with improved properties over PtOEP and PdOEP including significantly improved photostability (in [61] the absorbance of PtOEPK was found to decrease by only 12% after 18 hours of continuous UV illumination, while that of PtOEP was found to decrease by 90% under the same conditions), longwave emission, and good compatibility with Light-Emitting Diode (LED) excitation sources [99]. PtOEPK in particular has attracted much interest as an oxygen-sensitive probe. Its photostability has been found to be significantly (∼10 x) higher than that of PtOEP [51], making PtOEPK much more useful in intensity-based measurements and applications requiring long measurement times. Oxygen sensors using PtOEPK have been used in many applications, including glucose biosensors [11], microfluidics and microfluidic cell culture [48,54,55,78,100], inter- and intra-cellular measurements [39,51], food packaging [12], and other biological applications [56].

The aforementioned metalloporphyrin compounds are generally encapsulated in a polymer or sol-gel matrix (Section 4 discusses these matrices in more detail). Another class of commonly used metalloporphyrin compounds is water-soluble and generally bound to albumin compounds before use. These compounds include platinum (Pt) and palladium (Pd)-coproporphyrin [49,83,101,102], palladium *meso*-tetra-(4-carboxyphenyl) porphine [69,102–105] and the polyglutamic phosphorescent “Oxyphor” probes [106–120], all of which have mostly been used via intravenous injection for *in vivo* biological oxygen imaging. In addition to the water-soluble metalloporphyrins, there are also water-soluble ruthenium complexes, such as ruthenium tris(2,2′-dipyridyl) dichloride hexahydrate (RTDP) [71,95].

### 3.3. Summary and Applicability to Microfluidic Cell Culture

During microfluidic cell culture, the cells may be in contact with the probe molecule and oxygen sensor as a whole for extended periods of time, extending from hours to days. It is important that these materials be biocompatible, with no cytotoxic effects. O’Riordan *et al.* investigated indicator leaching into various simulated food components and found that the leaching of PtOEPK and Ru(dpp)2+ from polymer matrices into most aqueous solutions (with the exception of 95% ethanol) could not be detected [90]. No evidence of toxicity of the Oxyphor probes has been presented, with studies using Oxyphor R2 in rats at concentrations of up to 6.7 mg/kg body weight (∼40 μM in blood) showing no evidence of toxicity up to ten days after injection [121]. Dobrucki [122] found that Ru(phen)^2+^_3_ can have phototoxic effects. When used as a dye, repeated illumination of a sample caused the plasma membranes of cells to rupture, and the dye was observed to enter the cell nuclei and cytoplasm. This toxicity may be due to the generation of singlet oxygen when the Ru(II) complex is illuminated. Phototoxic effects were not detected for Ru(bipy)^2+^_3_ in the concentration range of 2 × 10^−4^ M.

Many of the oxygen-sensitive compounds are excitable with blue, green, yellow, or orange LEDs [60,61], offering a great advantage for small, ideally low-cost applications such as microfluidics. The sensitivities of the various ruthenium or metalloporphyrin compounds dictate the oxygen levels at which they are best used (for example, different sensors should likely be used for studying anoxic environments than those used for normal cellular environments or atmospheric conditions).

The water-soluble compounds do not present the same advantages for microfluidic systems as they do for *in vivo* biological imaging, where the possibility of injection of water-based dye solution facilitates less invasive imaging and even imaging through skin. In microfluidics, it may be desirable to use the microfluidic channel and cell culture setup more than once. In this situation integrating the sensor into the channel allows the indicator to be reused as well, potentially lowering the cost of the testing setup. For microfluidic cell culture applications, incubation times can be on the order of hours or even days, often requiring the circulation of fresh culture media over this time period. This application would require significantly more water-soluble luminescent indicator than would be required for a device-integrated sensor if all of the circulated solution is to be stained. Furthermore, encapsulating the sensor in a polymer or sol-gel matrix reduces the likelihood of unwanted interaction with the sample under test. Nevertheless, there are advantages (such as obtaining 3-D maps of oxygen distributions) to adding the indicator to the fluid in microfluidic channels, and this use has been previously demonstrated using RTDP [71,95]. Table 1 presents a summary of indicators in various encapsulation materials along with some of their properties.

## 4. Indicator Encapsulation Media

As mentioned previously, the luminescent indicator compound is often immobilized and encapsulated in a polymer or sol-gel matrix to improve sensor properties and reduce unwanted interaction with the sample under test. The encapsulation matrix can be patterned and holds the luminophore in place on the substrate. The encapsulation matrix has been found to greatly affect many of the properties of the oxygen sensor, such as its sensitivity and Stern-Volmer calibration function [125]. In particular, the oxygen diffusion constant of the polymer matrix is a very important parameter; it controls how easily the oxygen in the sample can migrate to the indicator compound and as a result greatly affects the sensitivity and response time of the sensor [37]. This section introduces some of the commonly used immobilization matrices and discuss their applications and potential for use in microfluidic cell culture. Further detail on encapsulation matrices in general can be found in [44,125–127].

### 4.1. Polymers

Several criteria need to be taken into consideration when choosing a polymer matrix for a luminescent oxygen-sensitive indicator. Aside from the aforementioned permeability to oxygen, the matrix’s mechanical stability is an important property in applications such as aquatic sediment mapping, however in microfluidic cell culture this property is often less important. If the sensor is patterned on the channel surface, the adhesion of the sensor and thus the polymer matrix to the channel should be sufficient such that microfluidic flow does not detach or damage the sensor. If the sensor is to be reused, the polymer matrix needs to be able to withstand whatever cleaning process is necessary. For microfluidic cell culture, the polymer matrix must be biocompatible. Finally, the chosen indicator needs to have good solubility in the matrix material in order to form homogeneous sensor films.

Commonly used polymers and corresponding references for their use in optical oxygen sensors include: polystyrene for [Ru(dpp)_3_]^2+^, PtOEPK, PdOEPK, and PtOEP indicators [11,12,28,38,46,48,52,54,55,59–61,78,90,91,100,125,128,129]; polymethyl methacrylate (PMMA) for PtOEP [60]; poly- decyl methacrylate (PDMA) for PtOEPK [56], polyvinyl chloride (PVC) for PtOEPK, PdOEPK, and [Ru(dpp)_3_]^2+^ [39,60,125]; ethyl cellulose for [Ru(dpp)_3_]^2+^ [38]; and silicones for PtOEP, [Ru(dpp)_3_]^2+^, and to encapsulate dye-adsorbed silica beads [38,47,53,62,130]. Additionally, working sensors have been created using [Ru(phen)_3_]^2+^ in photopatternable silicone [93].

Although the addition of plasticizers to polymer matrices such as PVC allow sensor properties such as response time and sensitivity to be optimized for applications of interest, their use can lead to significant changes from the ideally linear Stern-Volmer calibration equation of the resultant oxygen sensors [125].

### 4.2. Silica, Ormosil, and Sol-gel

Indicators such as [Ru(phen)_3_]^2+^ have been adsorbed to silica microbeads [38,47,130] and then either used as-is or encapsulated in silicone films. Organically modified silica (ormosil) and sol-gel particles and layers have also been developed and optimized in an effort to improve the properties of optical oxygen sensors [126]. Ormosils and sol-gels are very promising as encapsulation matrices, showing excellent optical and physical properties and good porosity/permeability to oxygen as well as the ability for the layer properties to be customized to various sensor applications [126,127]. Oxygen sensors using them have been developed [46,77,88,131–133] and used in various applications, including aquatic sediments [41,87].

Most of these commonly-used encapsulation media are applicable to microfluidic cell culture, and the best choice for a particular application depends on the indicator of interest, the desired level of sensitivity, and the desired sensor format. Previous applications in microfluidics have predominantly used polymer encapsulation matrices such as polystyrene [48,54,55,78,100] and poly(dimethylsiloxane) (PDMS) [53].

## 5. Oxygen Sensor Formats

**Figure 4.**
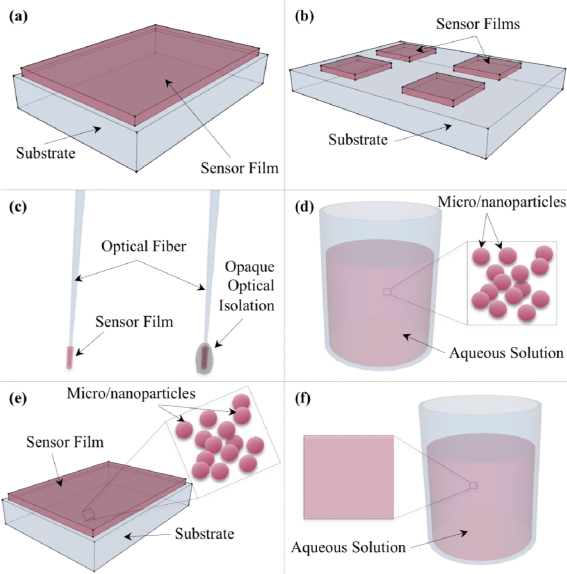
**(a)** Thin film sensor. **(b)** Patterned thin-film sensor. **(c)** Tapered optical fiber sensor without and with opaque polymer optical isolation (shown as partially transparent for figure clarity). **(d)** Micro/nanoparticle sensors suspended in aqueous media. **(e)** Micro/nanoparticle sensors suspended in a thin film. **(f)** Water-soluble sensor compound dissolved in aqueous media.

For microfluidic cell culture it is possible to use one of several oxygen sensor formats. Thin sensor films integrated into the cell culture device or substrate present perhaps the most obvious solution, but it may also be possible to integrate optical fiber-type sensors, micro/nanoparticle sensors, or even directly stain cells or the cell culture media with soluble oxygen-sensitive compounds. An illustrative overview of some of the sensor formats is presented in Figure 4. This section presents some of the previous work performed with these sensor formats and discusses how they may be applied to microfluidic cell culture.

### 5.1. Thin-film Sensors on Substrate

Thin-film type sensors are commonly used, and are generally fabricated by either pipetting or spinning solutions of the indicator and encapsulation medium onto a substrate of interest such as glass slides, polymers, or polyester foils. This type of sensor has been quite widely used as un-patterned films [28,38,46,47,50,62,63,77,91,130]. A similar process has also been used to create patterned layers by pipetting only small areas or performing a pipetting and lift-off process [11,12,54,55,61,78,90,125]. Fabricated thin layers have been lithographically patterned using PDMS “stamps” as masks in a dry etch process [48,100], and using a chromium mask layer [128]. Additionally, Ambekar *et al.* created photolithographically patterned thin-film oxygen sensors utilizing photopatternable silicone [93], however some difficulty was encountered with the use of a platinum porphyrin indicator, as it was highly absorbing at the wavelengths necessary to expose the photopatternable polymer.

Thin-film sensors are usually excited with either trans- or epi-illumination, but the excitation light has also been provided using optical fiber coupling [90] and evanescent fields from the glass substrate [50] or polymer waveguides [94].

Thin-film oxygen sensors have been integrated successfully with microfluidics [48,78,100] and used for microfluidic cell culture in order to monitor the dissolved oxygen concentrations during the culture of three types of bacteria requiring differing oxygen levels as well as mammalian cells [54,55].

### 5.2. Optical Fiber Sensors

Oxygen-sensitive micro-optodes are another commonly used sensor format, wherein the oxygen-sensitive dye and encapsulation matrix are attached to the end of an optical fiber. The optical fiber can provide the excitation light, carry the emitted luminescence to the detector, or both [36,39,60,86,87]. Layers of black silicone have been used to optically isolate the sensor film from its surroundings, and arrays of the sensors have been used to obtain oxygen concentration gradients [59]. The optical fiber has been pulled to fabricate tip diameters as small as 5–10 μm, but larger (10–40 μm) diameters are usually used to increase signal strength [59,86]. Other research has only used the optical fibers as a means of coupling the light to and from the sensor film, where the sensor layer is fabricated on a different substrate [90].

While the fiber optic platform presents a convenient method for coupling light to and from the sensor, integration with microfluidic devices is likely more difficult and inconvenient than that for the thin-film sensor platform. Nonetheless, there may be advantages to the fiber optic sensor platform, and integration with microfluidic cell culture should be possible. Similar to the fiber optic platform but possibly easier integrated with microfluidic cell culture, the ends of on-chip sol-gel waveguides have been coated with an Ru(dpp)_3_^2+^ compound encapsulated in sol-gel; this sensor platform was used to sense gaseous oxygen concentrations [133]. Although these waveguides are quite large (100 μm by 100 μm in cross-section), they lie parallel to the substrate and microfluidic cell culture environments could potentially be designed to incorporate them and bonded above them.

### 5.3. PEBBLE/Microparticle/Nanoparticle Sensors

The desire to create a versatile sensor platform with both the advantages of indicator encapsulation and the possibility of intracellular measurements led to the development of microparticle, nanoparticle, and “Probing Explorers for Bioanalysis with Biologically Localized Embedding” [56] or “Probes Encapsulated by Biologically Localized Embedding” [51] (PEBBLE) sensors. PEBBLE sensors are generally fabricated with the luminescent dye embedded in an ormosil matrix, and ratiometric, intensity-based measurements using these sensors have been used to map oxygen concentrations inside cells [51]. Other, PDMA-based ratiometric PEBBLEs have been used to monitor oxygen concentrations in human plasma [56]. Reviews of the applications of nano-sized PEBBLE sensors, including those for dissolved oxygen measurements, in biological and intracellular applications have been presented [134,135], and another review of various sensor technologies for monitoring various indicators of metabolic activity (again including dissolved oxygen PEBBLEs) inside cells has been written [14].

Other microparticle and nanoparticle oxygen sensors have been fabricated by doping polymer or silica beads with luminescent indicator dye [47,52,57] or by grinding indicator-doped ormosil [29]. These microparticle and nanoparticle sensors have been used directly [52] or embedded in another material such as silicone [47] or hydrogel [29] to form thin-film sensors. Microparticle and nanoparticle sensors could be integrated in the cell culture area by adding the particles to silicone or hydrogel thin-films within the channels.

### 5.4. Water-Soluble/Macromolecular Probes

The final general sensor platform is the dissolved, or macromolecular probe. This format uses water-soluble probes, which may be bound to albumin or other molecules to improve sensor characteristics. This probe format is versatile as it may be added to aqueous materials, including those for microfluidic cell culture. Water-soluble probes have been primarily used for *in vivo* biological imaging [49,52,83,102,103,105,107,108,136], but they could potentially be applied to other aqueous environments.

Water-soluble probes do suffer from several disadvantages. Because they are not encapsulated in a solid matrix, they are much more likely to interfere with their environment (e.g., binding to biological sample components or changing luminescence properties with changing sample chemical composition [52]) and it is more difficult to control the sensor parameters, such as its sensitivity and oxygen selectivity. As such, there has been effort to develop water-soluble probes that are encapsulated by or bound to other molecules to help overcome these disadvantages; dendritic encapsulation, whereby the luminophore is located inside a cage made up of repeatedly branched, large molecules (dendrimers) is one of the most promising of these methods [137].

Water-soluble probes could be used to monitor dissolved oxygen concentrations during microfluidic cell culture, as they could be added to the cell culture media to map oxygen concentrations in the entirety of the microfluidic channel. Using water-soluble probes could allow techniques such as tomographic imaging to map 3-D images of oxygen concentrations within the cell culture area. However, it is likely that a greater amount of potentially expensive probe molecules would be required for the water-soluble probe platform in comparison with the thin-film method, as the probes would need to be added to all of the cell culture solution and reuse may be impractical. Nevertheless, as mentioned previously, water-soluble RTDP has been applied to dissolved oxygen monitoring in microfluidic channels [71,95].

## 6. Optical Measurement Systems

The final main component in the design of optical oxygen sensors is the optical measurement system. This system consists of, at the minimum, a light source to excite the luminescent dye and a detector to detect the luminescence emission, and may also include an imaging system to increase the spatial resolution of oxygen measurements. This section gives an overview of some of the types of components previously used for oxygen sensing, with a focus on their usability for microfluidic cell culture.

### 6.1. Excitation Light Sources

The excitation light source needs to emit light in a spectrum compatible with the excitation spectrum of the luminescent indicator. Furthermore, it should not emit in the emission spectrum of the indicator. To prevent this, an excitation optical filter is commonly placed between the excitation source and the sensor, as was illustrated in Figure 1 and Figure 2. For lifetime detection the excitation source needs to be modulated, requiring the use of either a pulse-able source or an optical chopper.

Because LED sources are inexpensive and may be pulsed or modulated, they are very commonly used as excitation sources for optical oxygen sensors [11,12,28,36,38,46,50,54,55,59,60,62,63,78,87, 91,107,108,130], and excitation spectra which overlap well with LED emission spectra are considered an advantage of many indicator compounds such as Pt- and PdOEPK [61]. For excitation spectrum versatility and compatibility with other systems such as fluorescent microscopes, filtered broader-spectrum sources such as Xenon flash lamps and mercury-arc lamps have also been used [46–51,52,56,83,100–103,105,136]. Finally, laser excitation sources offer a very narrow emission spectrum, which often does not require any excitation filter [39,77].

Previous microfluidic oxygen sensors have used LED excitation [54,55,78], laser excitation [95], and filtered broad-spectrum excitation sources [48,53,71,100], and any of these could also be applied to microfluidic cell culture. It is, however often ideal to integrate the luminescent oxygen sensor measurement system with a fluorescence microscope or other optical system already in use. As such, the filtered broader-spectrum sources already used in fluorescence microscopes are ideal to be used as excitation sources, but they may be difficult to modulate. Other sources such as LEDs and lasers could potentially be integrated with many microscope systems as well.

### 6.2. Detectors

The detector used in the optical measurement system needs to be compatible with the emission spectrum of the luminescent dye and the measurement method (*i.e.*, intensity or lifetime), and a 2-D array of detectors can be used to image a spatial gradient in oxygen. Simple point detectors such as photodiodes [11,12,54,55,61,78,107] and photomultiplier tubes (PMTs) [36,52,59,60,83,87,101,102,105] are often used for emission detection in oxygen sensors due to their simplicity and fast response time, which is a particular advantage when used for lifetime detection.

A detector array is necessary for mapping oxygen concentrations in 2-D, which may be of interest in microfluidic cell culture applications. The most commonly used detectors for this application are CCDs [28,38,39,46–48,56,62,63,91,100,103,108,130,136], but phototransistor arrays [77] have also been successfully used.

Any of these detectors could be compatible with microfluidic systems for cell culture. Previous work with microfluidic oxygen sensors has mainly used photodiodes [54,55,78] and CCDs [48,53,71,95,100].

### 6.3. Imaging Systems

The final component of the optical measurement setup is the imaging system for 2-D images of oxygen distributions. The imaging system increases the flexibility of the oxygen sensing system, as it allows the spatial resolution of the oxygen images to be tuned as necessary by changing lenses or objectives. Some applications do not require imaging optics [77], but other applications (and likely microfluidic cell culture experiments) may require macro lenses or even complete microscope setups. As such, the ideal and most flexible solution if 2-D maps of oxygen distributions are required is the integration with a microscope or zoom lens. This integration is fairly straightforward and has been previously demonstrated with intensity-based sensing [39,47,48] and, while more difficult, has also been demonstrated with lifetime-based sensing [103,104,136].

## 7. Optical Oxygen Sensors in Microfluidic Cell Culture and Analysis

**Figure 5.**
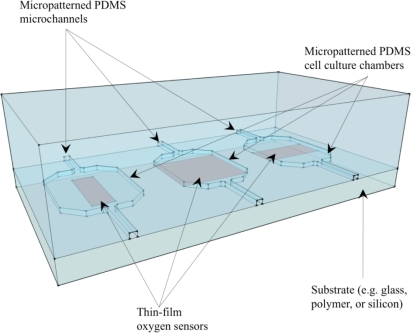
Illustration of enclosed PDMS microfluidic system for cell culture with possible designs for integrated optical oxygen sensors.

Microfluidic systems for cell culture can be fabricated through the technique of soft lithography, which involves casting PDMS structures from a photolithographically defined mold. The resulting transparent and biocompatible PDMS structure can form closed microfluidic channels and chambers when bonded to another substrate. PDMS is highly permeable to oxygen; the oxygen diffusivity (*D* = 4.1 × 10^−5^ cm^2^/s) and solubility (0.18 cm^3^ (STP)/cm^3^) permit passive permeation of oxygen through such devices for cell culture [71]. An example enclosed PDMS microfluidic system for cell culture with possible designs for integrated oxygen sensors is illustrated in Figure 5.

**Figure 6.**
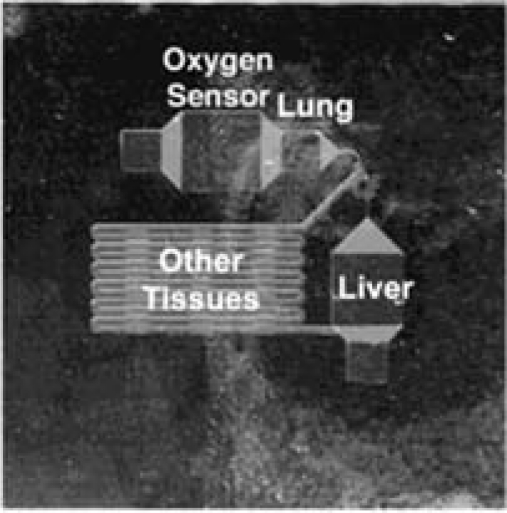
Photograph of the fabricated three-chamber microfluidic cell culture analog device with integrated optical oxygen sensor. Reprinted from [81] with permission from John Wiley and Sons.

Optical oxygen sensors have already been applied to microfluidic cell culture with very promising results. Sin *et al.* reported a three-chamber microfluidic cell culture analog device employing an optical dissolved oxygen sensor [81]. The device was used to culture three types of mammalian cells in interconnected chambers, forming a compact platform simulating animal testing for chemicals and pharmaceuticals. The integrated dissolved oxygen sensor enabled real-time readout of the oxygen levels in the circulating culture media. The oxygen sensor used a compound of Ru[dpp]_3_^2+^ immobilized onto resin particles, encapsulated in thin-film PDMS sensor patches on the substrate. Frequency-domain lifetime sensing was used, employing LED excitation and photodiode detection. The device as presented in the original journal paper [81] is presented in Figure 6. This work highlights some of the advantages that microfluidic platforms can bring to cell culture systems. The design permitted the culture of cells in three interconnected chambers which represented the lung, liver, and other tissue compartments in a pharmacokinetic model. Flow characteristics, including liquid residence times and shear stress on cells, were controlled to be within physiological values. The ability to measure oxygen within the design allowed Sin *et al.* to monitor gas exchange. By providing more realistic models for drug adsorption, distribution, and metabolism kinetics in pharmacological testing, further development of such systems can contribute to reducing the need for animal testing.

Sud *et al.* integrated optical dissolved oxygen sensors into microfluidic channels containing C2C12 mouse myoblasts to monitor the oxygen levels as a function of space and of cell density [95]. From the same group, Mehta *et al.* integrated optical dissolved oxygen sensors into a microfluidic bioreactor and took measurements upstream and downstream of adherent cells cultured in the microchannel, finding that the downstream oxygen concentration was highly dependent on cell density and fluidic flow rate [71]. Both of these works used the water-soluble oxygen indicator RTDP dissolved in the fluid pumped through the channels. Both intensity-based measurements and RLD-based lifetime imaging modalities were used. Although they used a high concentration of 1 mg/mL (approximately 1.3 mM) RTDP in order to obtain useful fluorescence signal with low exposure time, the presence of this dye in the culture media contributed to less than 10% of the cell death over the course of 5 hours during this work. However, longer incubation periods exceeding 1 day in this concentrated dye did decrease cell viability.

Mehta *et al.* [138] have also found that by using a combination of PDMS and rigid polymers in the construction of a perfusion cell culture system, lower oxygen tension can be achieved than in devices constructed entirely of PDMS. Using RTDP dissolved in solution, they verified that they could achieve oxygen concentrations as low as 1% using glycol-modified polyethylene terephthalate (PETG) channels bonded to flexible PDMS membranes. The flexible PDMS bottom permitted the use of deformation-based on-chip valving and pumping, while the relatively oxygen-impermeable, rigid PETG material permitted them to reach the low oxygen conditions which are needed for studies of embryonic and stem cell differentiation, ischemia, and cancer.

Lin *et al.* integrated several dissolved oxygen and glucose sensors along the length of a cell culture microchannel so as to quantify concentration gradients in the cells’ environment along the channel [53]. The sensors were fabricated using a ruthenium dye embedded in PDMS, which was used to fill microtrenches in the PDMS microchannel walls, and intensity-based measurements were used. The work found that both the oxygen and glucose concentrations in the channel were dependent on the fluidic flow rate; this was expected because the cultured cells’ oxygen and glucose consumption remained relatively constant while the supply of oxygen and glucose was altered by the change in flow rate.

**Figure 7.**
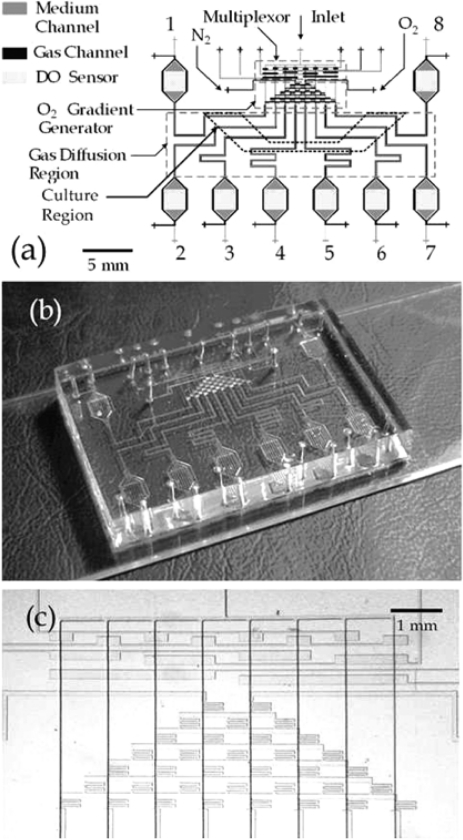
**(a)** Schematic diagram of the microfluidic oxygenator with integrated oxygen sensors. **(b)** Photograph of the fabricated oxygenator device. **(c)** Microscope image of the microfluidic multiplexor and oxygen concentration gradient generator. Reprinted from [55] with permission from the American Chemical Society.

Lam, Kim, and Thorsen have created a microfluidic oxygenator with an array of channels of differing oxygen concentrations for cell culture, employing an optical dissolved oxygen sensor integrated at the end of each microchannel. The oxygen gradient generator, which was comprised of one inlet for O_2_ and one for N_2_ gas followed by a network of mixing channels leading to a number of parallel outlet channels, yielded different dissolved oxygen concentrations in each outlet microchannel. Integrated PtOEPK-polystyrene film sensors permitted *in situ* measurement of these concentrations during cell culture. The device schematic diagram, fabricated device, and microscope image of the gradient generator are presented in Figure 7 [54]. Intensity-based imaging employing LED excitation and photodiode detection was used. This system has been used to culture mammalian cells as well as aerobic and anaerobic bacteria to investigate the effect of dissolved oxygen concentrations on the growth patterns of cells of differing oxygen requirements [54,55].

Oppegard *et al.* [139] used slides pre-coated with an oxygen-sensitive ruthenium complex (FOXY SGS; Ocean Optics) to study breast tumor cell migration. Fluorescence intensity measurements employing a fluorescence microscope and CCD detection were used to quantify the oxygen levels in the device in order to determine the effects of oxygen levels on tumor cell migration through a porous membrane. A parylene C coating on the highly oxygen-permeable PDMS was used to control the oxygen diffusion through the device, enabling the study of hypoxic oxygen levels. The study of tumor cell migration is of significant interest because it is related to cancer metastasis and studying its dependence on oxygen levels may help in the understanding of metastasis and the development of cancer treatments.

Finally, single cell oxygen consumption rates have been measured using optical oxygen sensors situated near single-cell traps. Cells trapped in microwell arrays [140] and in SU-8 negative epoxy photoresist micro-cups [141] were studied using patterned sensor rings formed from PDMS-encapsulated oxygen-sensitive microspheres and photopatternable SU-8-encapsulated platinum porphyrin indicator, respectively. Combining single-cell isolation and analysis with oxygen sensing as was accomplished in these works could potentially provide a useful tool for researchers studying cell metabolism and other phenomena at a single-cell level.

Each of these devices demonstrates a different method of integrating optical oxygen sensors with microfluidic cell culture or cell analysis, employing point measurements as well as measurements of 2-D gradients, lifetime and intensity-based measurements, dissolved as well as thin-film sensors, and a range of sensor compounds. The integration of optical oxygen sensors into each of these microfluidic cell culture devices facilitated real-time and *in situ* oxygen concentration measurements within compact, controllable, and functional microfluidic cell culture setups, which would not otherwise have been possible.

Microfluidic platforms which incorporate hydrogels for three-dimensional cell culture can mimic the tumor microenvironment. In future work, the combination of optical oxygen sensors for real-time imaging and the ability to pattern tumor cells within a microscale model of microvasculature can help identify the factors which contribute to angiogenesis [142,143].

## 8. Conclusions

Microscale techniques for cell biological assays are increasingly becoming validated and applied in biological laboratories. Microfluidic devices can give unique functionalities for cell-based assays including single-cell analysis, patterned three-dimensional cell cultures, and precise control over the culture microenvironment. Microfluidic systems promise to provide a simple, scalable tool to apply standardized protocols used in cellular response assays. Device features ranging from tens to hundreds of microns will allow tracking and manipulation of tens to hundreds of cells, providing the ability to analyze small cell populations which is not possible using current standard techniques. Integration of sensing capability will increase their ease of use and the types of readouts that can be obtained. There is a very wide range of optical oxygen sensors that are compatible with microfluidic cell culture, and certain sensor types have already been successfully applied to this field. Thus, the integration of on-chip oxygen sensors with microfluidic cell culture and analysis platforms will provide a powerful tool which promises to have a large impact in drug discovery, quantitative biomedical sciences, and the development of novel therapeutics. The choices of sensing mechanism, luminescent indicator, encapsulation matrix, sensor format, and optical imaging system are highly interdependent and also highly dependent on the oxygen levels, measurement requirements, and existing imaging system of the chosen application. Future work may see new types of optical oxygen sensors seamlessly integrated with existing microfluidic cell culture equipment, allowing for simultaneous measurement of 2-D or even 3-D oxygen distributions along with other properties of interest. These measurements may facilitate the discovery of new correlations between these properties and oxygen levels.

## Figures and Tables

**Table 1.** Properties of indicator materials in various encapsulation matrices as previously reported.

**Indicator**	**Encapsulation Matrix**	**Unquenched Lifetime (μs)**	**Quantum Yield**	Reported Sensitivity*	**Excitation Peaks (nm)**	**Emission Peaks (nm)**	**[Refs]**

							
([Ru(dpp)_3_]^2+^	Polystyrene	5	NR	22% signal decrease from N_2_ to air	450	600	[60]
							
([Ru(dpp)_3_]^2+^	Plasticized PVC	5	NR	50% signal decrease from N_2_ to air	450	600	[60]
([Ru(dpp)_3_]^2+^	None	6.3 at 23 °C (silicone-soluble ion pair in 2-butanone )	0.3 (in water/ethanol) 0.35 (silicone-soluble ion pair in 2-butanone )	k_Q_(dissolved O_2_) = 2.5 (10^9^dm^−3^ mol^−1^s^−1^) (in methanol)	460	613, 627	[37,92]
([Ru(phen)_3_]^2+^	None	0.74 at 23 °C (silicone-soluble ion pair in 2-butanone )	0.08 (silicone-soluble ion pair in 2-butanone )	k_Q_(dissolved O_2_) = 4.2 (10^9^dm^−3^ mol^−1^s^−1^)	447,421	605, 625	[37,92]
[Ru(Ph_2_phen)_3_]^2+^	Sol-gel silica	5.8	NR	τ _N2_/τ_O2_ = 5	NR	NR	[123]
([Ru(bpy)_3_]^2+^	None	0.6	0.042	k_Q_(dissolved O_2_)=3.3 (10^9^dm^−3^ mol^−1^s^−1^)	423, 452	613, 627	[37,83]
([Ru(bpy)_3_]^2+^	Sol-gel silica	1.26	NR	τ _N2_/τ_O2_ = 2	NR	NR	[123]
**Indicator**	**Encapsulation Matrix**	**Unquenched Lifetime (μs)**	**Quantum Yield**	Reported Sensitivity *	**Excitation Peaks (nm)**	**Emission Peaks (nm)**	**[Refs]**

PtOEPK	Polystyrene	61.4 at 22°C	0.12	High	398, 592	759	[61]
PtOEPK	PDMA	NR	NR	Q_DO_ = 97.5%	NR	754	[56]
PdOEPK	Polystyrene	480 at 22°C	0.01	Very high	410, 602	790	[61]
PtOEP	Polystyrene	94.7 at 20°C	NR	τ_0_/τ_air_ = 3.60	383, 535	647	[60,98]
Pd-coproporphyrin	None (aqueous solution)	530 (no BSA), 1200 (BSA)	0.2	k_Q_ = 195 mmHg^−1^s^−1^	393. 545	667	[83,124]
Pt-coproporphyrin	None (aqueous solution)	100	0.4	NR	380, 535	650	[124]
Pd-*meso*-tetra-(4-carboxy- phenyl) tetrabenzoporphyrin-dendrimer (Oxyphor G2)	None (BSA solution at pH 6.8, 23.5 °C)	276	0.12	k_Q_ = 195 mmHg^−1^s^−1^	442, 632	800	[106,109]
Pd-*meso*-tetra-(4-carboxyphenyl) porphyrin-dendrimer (Oxyphor R2)	None (BSA solution at pH 6.8, 23.5 °C)	738	0.1	k_Q_ = 270 mmHg^−1^s^−1^	415, 524	700	[109]
Pd-*meso*-tetra (4-Carboxyphenyl) Porphine (Oxyphor R0)	None (albumin solution at pH 6.8, 23 °C)	705	0.06	k_Q_ = 246 mmHg^−1^s^−1^	416, 523	687	[106]

NR: Not Reported.

* Different measures of sensitivity were reported in different papers, and the values quoted in this table were those reported in the reference.
